# Novel screening model for infectious mononucleosis in febrile pediatric patients using a 3D-DIFF scattergram

**DOI:** 10.1186/s12879-025-11662-3

**Published:** 2025-11-07

**Authors:** Zhaoyang Peng, Jingxian Zhang, Lin Chen, Kesheng Li, Fengqing Cai, Wenbo Zheng, Lisu Huang, Baohai Chen, Jing Zou, Chunda Wang, Xiaoxia Gao, Bin Huang, Hongqiang Shen, Gang Yu

**Affiliations:** 1 Department of Laboratory Medicine, Children’s Hospital Zhejiang University School of Medicine, Hangzhou, 310052 China; 2 Clinical Department (IVD), Shenzhen Mindray Bio-Medical Electronics Co., Ltd, Shenzhen, 518057 China; 3 Department of Infectious Disease, Children’s Hospital Zhejiang University School of Medicine, Hangzhou, 310052 China; 4 Department of Data and Information, Children’s Hospital Zhejiang University School of Medicine, Hangzhou, 310052 China; 5 Sino-Finland Joint AI Laboratory for Child Health of Zhejiang Province, Hangzhou, 310052 China; 6 Department of Laboratory Medicine, Northwest Women’s and Children’s Hospital, Xi’an, 710061 China

**Keywords:** Blood cell parameters, Infectious mononucleosis diseases, Machine learning, Febrile pediatric patients

## Abstract

**Background:**

Infectious mononucleosis (IM) is an acute self-limited disease caused mainly by Epstein-Barr virus (EBV) that is prone to be missed or misdiagnosed in febrile children due to a lack of obvious clinical symptoms. Establishing a model based on blood cell parameters for identifying patients with IM is important for the subsequent diagnosis and treatment of children with fever.

**Methods:**

From January to December 2023, data of a total of 5511 febrile children (including 518 IM patients with primary EBV infection) were collected from Children's Hospital Zhejiang University School of Medicine. After assessing the changes in lymphoid clusters on the 3D-DIFF scatter plot and further analyzing the parameters of the scattergrams for quantifying the fluorescence signal changes in IM patients through the probability density function, eleven features were selected from among 41 blood cell parameters to construct the final Novel IM screening model (Novel IMs model).

**Results:**

The Novel IMs model performed well in three sets, screening IM patients with an accuracy of more than 91.92%. Compared others, the Novel IMs model had the best identification accuracy, specificity, and sensitivity and reached an area under the receiver operating characteristic (ROC) curve (AUC) in identifying IM patients of 0.96.

**Conclusion:**

This study is the first to develop a machine learning model to screening IM patients from febrile children using parameters obtained from blood cell scattergrams. This convenient and low-cost technique for initial screening of IM by hematology analysis may improve the screening coverage of the disease and further reduce misdiagnosis and underdiagnosis.

**Supplementary Information:**

The online version contains supplementary material available at 10.1186/s12879-025-11662-3.

## Introduction

In the absence of specific clinical symptoms or clear causes, establishing a definite diagnosis for children with fever is often a challenge for primary care physicians, emergency department clinicians and pediatricians [1]. Infectious mononucleosis (IM), an acute infectious disease caused mainly by the Epstein‒Barr virus (EBV) [2], is difficult to diagnose in febrile children, mainly because the diverse clinical symptoms of IM, especially those that are nonspecific or absent in children, are prone to being missed by doctors [2–4]. Children under 5 years old with IM usually present with fever, pharyngitis and lymphadenopathy, and they are more likely than adults are to have upper respiratory symptoms [5, 6]. As a result, 53.10 ~ 72.69% of patients with IM are misdiagnosed with upper respiratory tract bacterial infections and receive antibiotic treatment [7, 8]. In addition, severe complications of the disease, such as splenic rupture or hemophagocytic lymphohistiocytosis, may be life-threatening [4, 6]. Therefore, accurate diagnosis of IM in febrile children is very important.

The clinical diagnosis of IM is usually confirmed by the presence of clinical symptoms and laboratory tests. The heterophilic antibody test is specific for IM, but it also has a high false-negative rate in children under 5 years old [9, 10]. Some studies have suggested that if the heterophile antibody results are negative, the EBV test should be performed in patients with an increased proportion of lymphocytes or reactive lymphocytes to comprehensively diagnose IM [4]. However, the antibody tests are costly, and patients with nonspecific clinical symptoms or who are asymptomatic may not be presented with the opportunity to undergo these antibody tests. In febrile children, blood cell analysis is needed to preliminarily determine the cause of the fever. Previous studies have shown that for blood cell analysis, the diagnostic specificity for IM when the lymphocyte proportion is greater than 50% reaches 93%, but the sensitivity is only 56% [11, 12]. These results indicate that the lymphocytes in IM patients are altered, but the proportion alone is insufficient to fully represent this alteration. The use of artificial intelligence (AI) to mine the changes of lymphocyte clusters of suspected IM patients from the 3D-DIFF scatter may hold promise for effectively reducing the missed diagnosis or misdiagnosis rate for IM. Recent studies have used lymphocyte scatter plots to indicate the presence of apoptotic cells in IM, which are the result of degenerative changes in virus-infected B lymphocytes [13]. These findings provide clues for IM screening [14, 15], but apoptotic cells are not a specific manifestation of IM. 3D-DIFF (blood count with differential counting of white blood cells) scatter plots derived from data obtained from advanced hemocytometers contain a wealth of information. This study used AI to mine the features of 3D-DIFF scatter plots derived from peripheral blood to establish a machine learning (ML) model to identify IM patients from febrile children.

## Methods and materials

### Patient

This retrospective study included the data of patients under 14 years old with fever (axillary temperature > 37.5 °C) who visited the fever clinic of the Children's Hospital Zhejiang University School of Medicine from January to December 2023. All enrolled subjects underwent complete blood count (CBC) tests and laboratory examinations for auxiliary diagnostic purposes. We also collected sex and age information from the enrolled subjects. The exclusion criteria were as follows: 1) immunodeficiency or immunosuppression; 2) lack of CBC or other laboratory test results; 3) long-term use of any drugs that may affect the results of the CBC; 4) antiviral treatment before the first visit to the clinic; and 5) previous EBV infection. Healthy control samples (*n* = 20) were used solely as visual references for 3D-DIFF scatterplot patterns during exploratory analysis and were not included in model development. This study was approved by the Ethics Committee of the Children’s Hospital, Zhejiang University School of Medicine (ethics batch number: 2022-IRB-268). Informed consent was waived because the research utilized only de-identified residual samples obtained during routine clinical testing, and the study posed no additional risk to patients.

### Laboratory examinations

#### Blood cell analysis

Venous or peripheral blood was collected from all enrolled patients for blood cell analysis using a Mindray BC-7500 CRP hematology analyzer (Mindray, Shenzhen, China) into EDTA-K_2_ blood collection tubes. The BC-7500 CRP hematology analyzer provides 75 parameters. First, we performed manual screening to eliminate uncommonly used channels and parameters with linear relationships with each other, ultimately yielding 41parameters, including white blood cell count (WBC#), percentages of lymphocytes (Lym%), neutrophils (Neu%), and monocytes (Mon%), red blood cell count (RBC#), hemoglobin concentration (HGB), platelet count (PLT#) and research parameters related to reactive lymphocytes, such as the percentage of high fluorescent cells (HFC%). It also includes the neutrophil-to-lymphocyte ratio (NLR), the platelet-to-lymphocyte ratio (PLR).

Fifteen IM patients and 15 patients with other viral infections were randomly selected from the cohort. The slides of these patients were stained with Wright's-Giemsa staining solution (Zhuhai Beisuo, China) using an SC-120 automatic slide maker and stainer (Mindray, China). The morphological results were analyzed with an MC-80 automatic digital cell morphological analyzer (Mindray, China), and the results were reviewed by morphological experts.

#### Other laboratory tests

The sera of all IM patients were collected for EBV antibody analysis (EBEA IgM, EBNA IgG, EBVCA IgG, and EBVCA IgM) (Shenzhen YHLO, China) with an iFlash 3000 chemiluminescence immunoassay analyzer (Shenzhen YHLO, China) and an EBEA IgG test (Beijing Beier, China). According to the diagnostic recommendations for IM [2, 16], the clinical symptoms and signs of the patient and the clinical diagnosis of the physician were considered to diagnose patients with EBNA-IgG (-), EBVCA-IgM (+), EBVCA-IgG (±), EBEA-IgG (-), and EBEA-IgM (-)with acute IM caused by a primary EBV infection, while those with EBNA-IgG (-), VCA-IgM (±), VCA-IgG (±), EBEA-IgG (+), and EBEA-IgM (±) were diagnosed with nonacute IM caused by a primary EBV infection.

All patients with upper respiratory tract infections included in this study underwent relevant laboratory tests. Patients were diagnosed with bacterial infection if their samples contained elevated C-reactive protein levels (Shenzhen Mindray, China) and were negative for EBV antibodies and other viral nucleic acids according to the results from the BC-7500 CRP (Shenzhen Mindray, China) as well as on the basis of their clinical symptoms and signs and the clinical diagnosis of the doctor. Throat swabs were collected from some patients and analyzed with multiplex reverse transcription PCR and capillary electrophoresis using an Applied Biosystems 3500 Dx gene sequencer (Thermo Fisher, USA) in conjunction with 13 respiratory pathogen multiplex kits (Health Genetech, China), including those for tested for influenza A viruses (H7N9, H1N1, H3N2, H5N2), influenza A virus H1N1 (2009), seasonal H3N2 virus, influenza B virus (Victoria and Yamagata lineage), adenovirus (ADE) (groups B, C, and E), Bocavirus, rhinovirus, parainfluenza virus (types 1, 2, 3, and 4), coronaviruses (229E, OC43, NL63 and HKU1), respiratory syncytial virus (RSV) (groups A and B), metapneumovirus, *Mycoplasma pneumoniae* and Chlamydia (*Chlamydia trachomatis* and *Chlamydia pneumoniae*). Viral or mycoplasma infection was confirmed according to the clinical symptoms of the patient, a negative EBV antibody test, and the doctor’s clinical diagnosis. Throat swabs collected from some patients were tested for severe acute respiratory syndrome coronavirus 2 (SARS-CoV-2) with the Applied Biosystems 3500 Dx gene sequencer and a coronavirus disease 2019 (COVID-19) nucleic acid detection kit (Adagene, USA). Viral infection was confirmed according to the patient’s clinical symptoms, a negative EBV antibody test, and the clinical diagnosis.

Patients with acute lymphocytic leukemia (ALL) were diagnosed and typed based on the cytomorphology, immunology, cytogenetics and molecular biology (MICM) diagnostic model adopted by the WHO [17].

#### Machine learning model

##### Data preprocessing and dataset partitioning

Data preprocessing included data cleaning—including the removal of duplicate data and erroneous inputs in the test samples to ensure data validity—and feature normalization to eliminate differences in feature distribution caused by inconsistences in the gain and calibration coefficients of different instruments and to avoid interference from outlier values during model training. Moreover, the units of the different features were normalized to the same dimensions to prevent the model from ignoring key parameters due to large dimensional differences. The standard deviation was used to normalize each characteristic parameter into a normal distribution with a mean value of 0 and a standard deviation of 1.

##### Feature screening and model evaluation

Using recursive feature elimination based on cross-validation, the area under the receiver operating characteristic (ROC) curve (AUC) of the models constructed from the 41 different feature parameters was calculated to determine the best feature combination and the optimal model Multiple machine learning methods were selected to identify optimal features and construct the models, including logistic regression (LR), support vector machine (SVM), linear discriminant analysis (LDA), and random forest classifier (RFC). The principles underlying these models are described below [18].

LR: The logistic function is used to convert the output of linear regression into a probability value, after which the model parameters are calculated on the basis of the maximum likelihood estimation so that the difference between the predicted probability and the true label is minimized [18].

SVM: The optimal hyperplane that best separates data corresponding to different categories is determined. Then, for a particular predicted category, the farther the distance from the calculated hyperplane, the more likely the sample data are to belong to this category [18].

LDA: The dimensionality of high-dimensional data are reduced such that after the data are projected onto a straight line, the dispersion of samples of different categories is guaranteed to be the maximum. The LDA model yields the class into which the predicted data are most likely to be classified according to the posterior probability [18].

RFC: An ensemble machine learning method that combines multiple decision tree models, yields the prediction results of all the decision tree models, and votes on the prediction results to produce the final classification prediction of the model [18].

##### Novel IMs model training and tuning

According to the TRIPOD guidelines [19], on the basis of the selected best feature combination and the optimal model (RFC), the prebuilt Novel IM screening (IMs) model in this paper was trained and tuned. Because of the imbalanced distribution of the number of IM and other samples in the training set, a weighting coefficient for the number of samples was included when training the model to ensure that equal attention was paid to both groups of samples.

During the model tuning process, the hyperparameter grid search algorithm was used to further tune the model. The predefined hyperparameter search list for the RFC model included regularization parameters, kernel function type, and kernel function coefficients. The model was constructed by iterating over the given hyperparameter combinations, and the optimal hyperparameters were selected by comparing the performance of the models constructed under each hyperparameter combination following fivefold cross-validation in the validation set. After completing the model construction, the model was calibrated with Platt Scalling.

##### Traditional parameter-based model construction

On the basis of conventional blood cell analysis parameters used in the field, training and tuning were performed in the same manner as the Novel IMs model to construct a Traditional IMs model whose performance was then compared with that of the Novel IMs model.

The dataset in this study was randomly divided into a training set (2205 patients), a validation set (2204 patients), and a test set (1102 patients) at a ratio of 4:4:2. The median (interquartile range) is used to describe continuous variables. The nonparametric Mann‒Whitney U test was used for statistical comparisons, and a *p* value < 0.05 was considered to indicate statistical significance. To demonstrate the performance of the model, first, we used the positive predictive probability to fully demonstrate its predictive capabilities for IM patients in the three datasets. Second, we compared the Novel IMs model with the parameters associated with the diagnosis of IM (Lym% and NLR) and Traditional IMs model used in previous studies by calculating the AUC, Decision curve analysis (DCA), sensitivity, and specificity and identifying the number of the false-positive and false-negative samples for each model with confusion matrices. The Novel IMs model was further explained via SHapley Additive exPlanations (SHAP) analysis. In addition, the Novel IMs model was used to determine the threshold for identifying patients with acute IM. This study used Python 3.7 scikit-learn for statistical analysis.

## Results

### Participant characteristics

A total of 5511 febrile children (54.04% boys, 45.96% girls) were included in this study (Table 1), of whom 518 had IM (54.05% boys, 45.95% girls). Additionally, 4888 patients with respiratory infection and 110 with acute lymphocytic leukemia (ALL) were included in the non-IM group in this study (male, 48.48%; female, 51.82%). The respiratory infection patients included those with different pathogens, among whom those with bacterial infections accounted for the highest proportion (1393 patients, 25.28%). The ages ranged between 2.0 and 7.0 years old, with a median of 5 years. The age distributions of the different groups were different, which is related to the differences in the incidence of the different diseases across different age groups. The patients in the RSV infection group were the youngest, as expected given the susceptibility of infants and young children to the disease [20]. The age distribution of patients in the IM group was between 3.0 and 7.0 years, with a median of 5 years.

**Table 1:** Characteristics of participants

Participants	n (%)	Male, n (%)	Female, n (%)	Age (years)	WBC (10^9^/L)	Lym%	Neu%	PLT (10^9^/L)	RBC (10^12^/L)
respiratory infection									
respiratory syncytial virus infection	1008 (18.29)	586(58.13)	422(41.87)	0.00 (0.00, 2.00)	6.86 (5.14 ~ 9.56)	57.65 (50.48 ~ 66.30)	31.50 (23.40 ~ 39.82)	304.00 (225.00 ~ 422.00)	4.37 (4.05 ~ 4.61)
influenza A virus infection	80 (1.45)	44 (55.00)	36 (45.00)	6.00 (4.00, 9.00)	7.17 (5.05 ~ 8.96)	21.40 (15.30 ~ 32.40)	68.35 (56.62 ~ 74.62)	249.00 (216.50 ~ 298.25)	4.52 (4.29 ~ 4.78)
influenza B virus infection	34 (0.62)	16 (47.06)	18(52.94)	5.00 (2.25, 7.00)	6.42 (5.02 ~ 7.52)	29.70 (20.60 ~ 42.97)	55.15 (44.98 ~ 63.42)	253.50 (182.00 ~ 314.75)	4.59 (4.26 ~ 4.84)
COVID-19 infection	50 (0.91)	27 (54.00)	23(46.00)	1.50 (1.00, 3.75)	6.82 (5.65 ~ 8.39)	32.00 (16.80 ~ 41.05)	58.55 (43.67 ~ 70.22)	236.50 (196.75 ~ 283.75)	4.60 (4.26 ~ 4.89)
adenovirus infection	1318(23.92)	715 (54.25)	603(45.75)	2.00 (0.00, 4.00)	7.59 (5.48 ~ 10.77)	44.60 (27.20 ~ 60.50)	45.05 (28.92 ~ 62.60)	287.00 (227.25 ~ 377.25)	4.37 (4.09 ~ 4.62)
bacterial infection	1393(25.28)	741 (53.19)	652(46.81)	4.00 (2.00, 6.00)	8.81 (6.56 ~ 12.20)	33.00 (22.80 ~ 45.50)	56.60 (44.20 ~ 68.80)	337.00 (264.00 ~ 431.00)	4.38 (4.16 ~ 4.63)
mycoplasma pneumoniae infection	1000 (18.15)	516(51.60)	484(48.40)	7.00 (4.00, 9.00)	7.58 (6.01 ~ 9.72)	29.00 (21.30 ~ 37.40)	61.10 (53.00 ~ 70.22)	308.00 (243.75 ~ 399.00)	4.46 (4.19 ~ 4.69)
acute lymphoblastic leukemia	110 (2.00)	53 (48.18)	57 (51.82)	7.00 (4.00, 9.00)	3.89 (2.50 ~ 7.33)	45.85 (26.68 ~ 83.80)	41.75 (10.27 ~ 56.08)	166.00 (68.00 ~ 268.50)	3.19 (2.52 ~ 3.74)
infectious mononucleosis	518 (9.40)	280 (54.05)	238(45.95)	5.00 (3.00, 7.00)	11.82 (8.80 ~ 15.66)	71.50 (62.20 ~ 77.60)	22.80 (17.00 ~ 31.20)	239.00 (189.00 ~ 291.75)	4.43 (4.18 ~ 4.63)
total	5511	2978(54.04)	2533(45.96)	5.00 (2.00, 7.00)	7.96 (5.99 ~ 10.53)	42.80 (28.10 ~ 58.10)	47.20 (31.88 ~ 62.10)	239.00 (237.00 ~ 388.00)	4.42 (4.15 ~ 4.66)

The basic blood cell analysis parameters (such as WBC#, Lym%, Neu%, RBC#, and PLT#) of each group are shown in Table 1. The increase in the number of white blood cells was the most significant in the IM group, followed by the bacterial infection group. The highest increase in proportion of lymphocytes was observed in the IM group, followed by the RSV infection group. In contrast, the proportions of neutrophils in the IM group and the RSV infection group decreased. The platelet count and red blood cell count were significantly decreased only in the ALL group.

#### D-DIFF scattergram characterization of IM patients

**Fig. 1:**
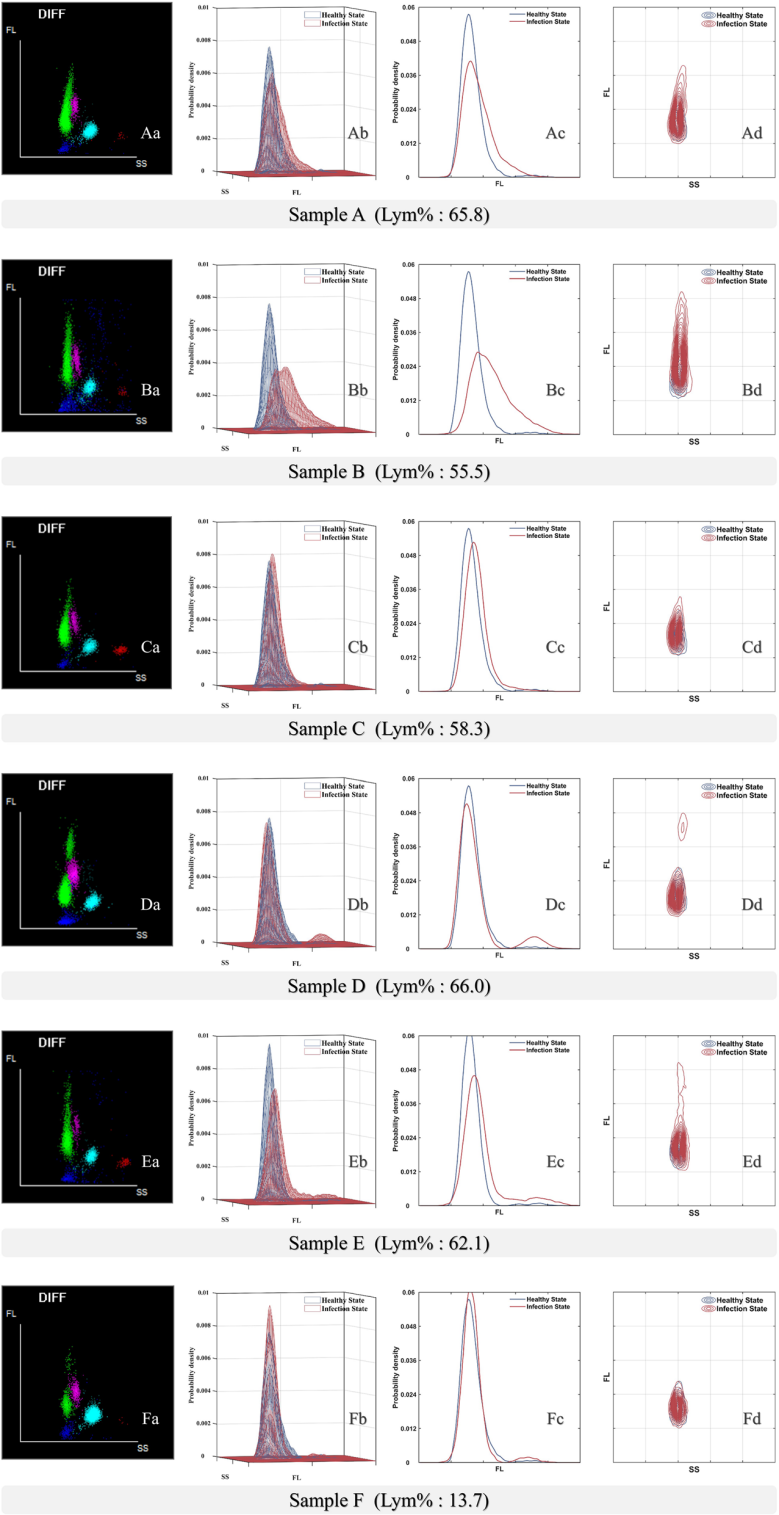
Sample **A**, **B**, **C** from IM group and Sample **D**, **E**, **F** from Non-IM group. **A**-**F**: Healthy controls (shown only as reference patterns for FL distribution comparison). Aa-Fa shows the scattergram of the SS-FL dimension in the DIFF channel of the patient samples in the BC-7500 CRP. Ab-Fb shows the 3-dimensional plot of the probability density distribution of the SS-FL dimension of the patient's lymphoid clusters. Ac-Fc shows the distribution curve of the patient's lymphoid clusters in the FL dimension. Ad-Fd shows the probability density distribution of the patient's lymphoid clusters in the SS-FL dimension. Notes: DIFF: blood count with differential counting of white blood cells, SS: side laser scatter cellular analysis, FL: Fluorescence

An elevated lymphocyte percentage is a nonspecific laboratory indicator in the diagnosis of IM [6], but this value alone could not be used to differentiate IM samples from non-IM patient samples. As shown in Fig. 1, five of the six samples presented with elevated lymphocyte counts. Although the percentages of lymphocytes were similar, the scatter plot shows that the distributions of Fluorescence (FL) in the lymphoid clusters of the IM patient samples were different from those of the other groups. By separating such samples, we can determine the FL distribution characteristics of the lymphatic clusters of IM patients. To better quantify the FL signal changes in IM patients, the distribution of all the cells in the 3D-DIFF scatter diagram in the FL direction was converted to the probability density of the entire lymphocyte cluster within the signal intensity range at the 3D level. As shown in Formula (2.1), the influence of the number of sample cells could be ignored in this way to allow focusing on only the distribution of lymphocyte clusters at each point. Subsequently, the changes in lymphocyte clusters from the healthy state to the diseased state were analyzed.

As shown in Fig. 1 Ab, Bb and Cb, compared with the lymphocyte clusters of healthy individuals, the lymphocyte clusters of the IM group as a whole migrated toward the high-FL area; more obvious migrations were accompanied by more obvious changes in the center of gravity of the cluster. As shown in Fig. 1 Ac, Bc and Cc, the distribution of lymphoid clusters in healthy individuals was concentrated in the low-FL area. The lymphoid clusters of the IM group were scattered in both the low-FL area and the high-FL area with no obvious preference. As shown in Fig. 1 Dc and Ec, most of the lymphoid clusters of the samples from the non-IM group were concentrated in the low-FL area, and a small portion was scattered in the high-FL area. To quantify the distribution width, change in the center of gravity and degree of dispersion of the lymphocyte clusters in the IM group, we determined the lymphocyte FL distribution width (D-Lym-SFL-W), center of gravity (D-Lym-SFL-P) and coefficient of variation (D-Lym-SFL-CV), as shown in Formulas (2.2), (2.3), and (2.4).

The lymphocyte proportions were similar in the IM group (samples A, B and C) and the non-IM group (samples D and E), and the distributions of lymphoid clusters were both discrete; however, the FL distributions of the D and E samples in the non-IM groups were in two areas (Fig. 1 Dd and Ed). We can quantify this finding by the peak-to-valley ratio of lymphocytes in the FL distribution (D-Lym-Peak-Valley-R), which can be used to differentiate the IM group and the Non-IM group. The larger the peak-to-valley ratio is, the more obvious the clustering is. The specific calculation method is shown in Formula 2.5.

**Fig. 2:**
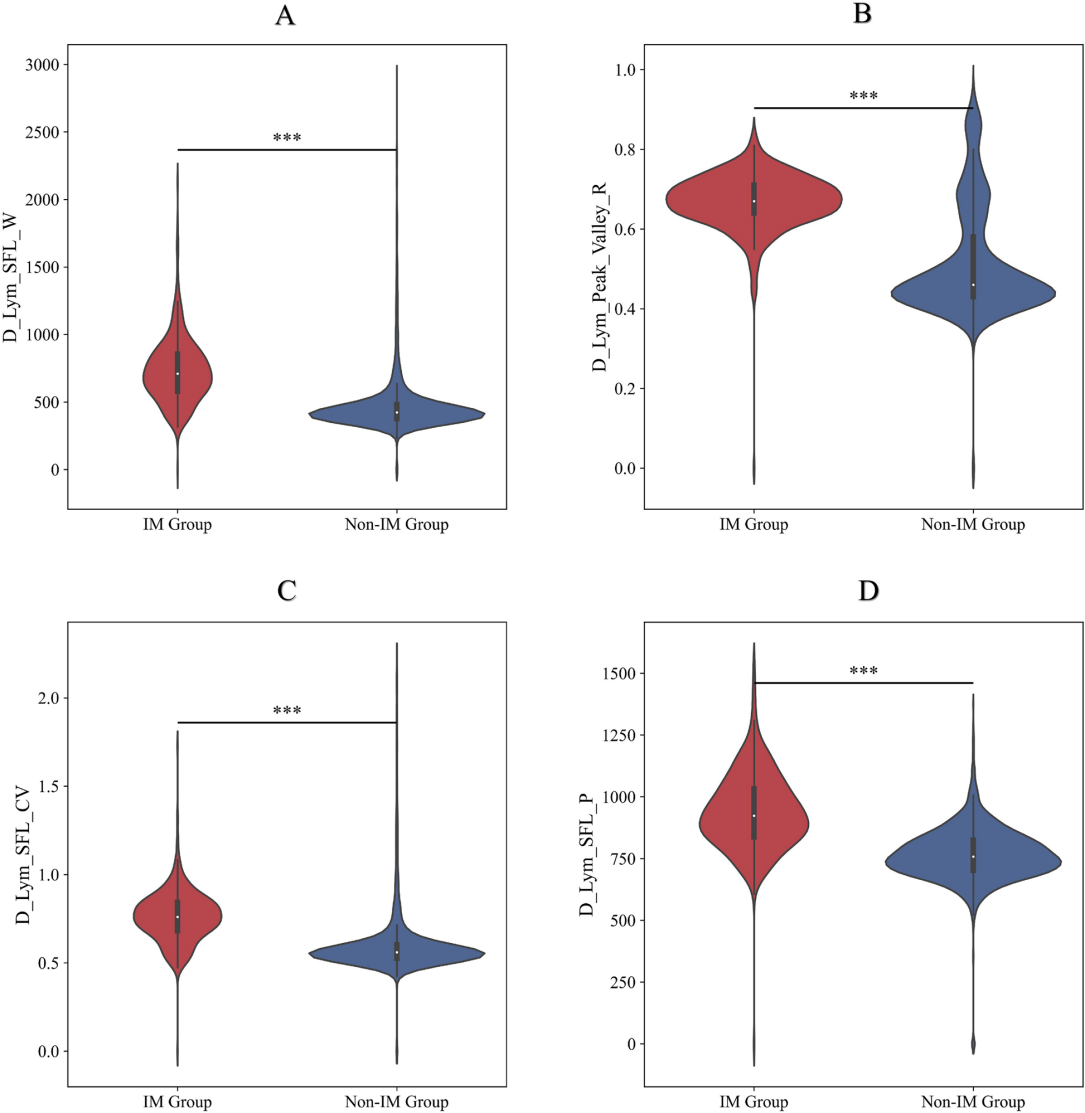
Mann‒Whitney U test to analyze scattergram parameters of the blood cells in the IM and Non-IM group. *p < 0.05, **p < 0.01, ***p < 0.001. Note: D-Lym-SFL-W: the lymphocyte FL distribution width, D-Lym-SFL-P: the lymphocyte FL center of gravity, D-Lym-SFL-CV: the lymphocyte FL coefficient of variation, D-Lym-Peak-Valley-R: the peak-to-valley ratio of lymphocytes in the FL distribution

The D-Lym-SFL-W, D-Lym-SFL-P, D-Lym-SFL-CV, and D-Lym-Peak-Valley-R of the two groups were compared (Fig. 2). The results showed that all three variables were significantly different between the IM group and the Non-IM group (*p* < 0.05).

2.1 $${Pd}_{SS,FL}={Cel{l}_{Num}}_{SS,FL}/Cell\_Num\_All$$

2.2 $$D\_Lym\_SFL\_W=Up-Down$$

2.3 $$D\_Lym\_SFL\_P=\sum_{i=1}^{FL-max}{FL}_{i}*{PD}_{i}$$

2.4 $$D\_Lym\_SFL\_CV=\sqrt{\frac{1}{n}\sum_{i=1}^{n}{\left({PD}_{i}-\overline{P }D\right)}^{2}/\left(\frac{1}{n}\sum_{i=1}^{n}{PD}_{i}\right)}$$

2.5 $$D\_Lym\_Peak\_Valley\_R=Peak\_Hei/Valley\_Hei$$

### Selected features

**Fig. 3:**
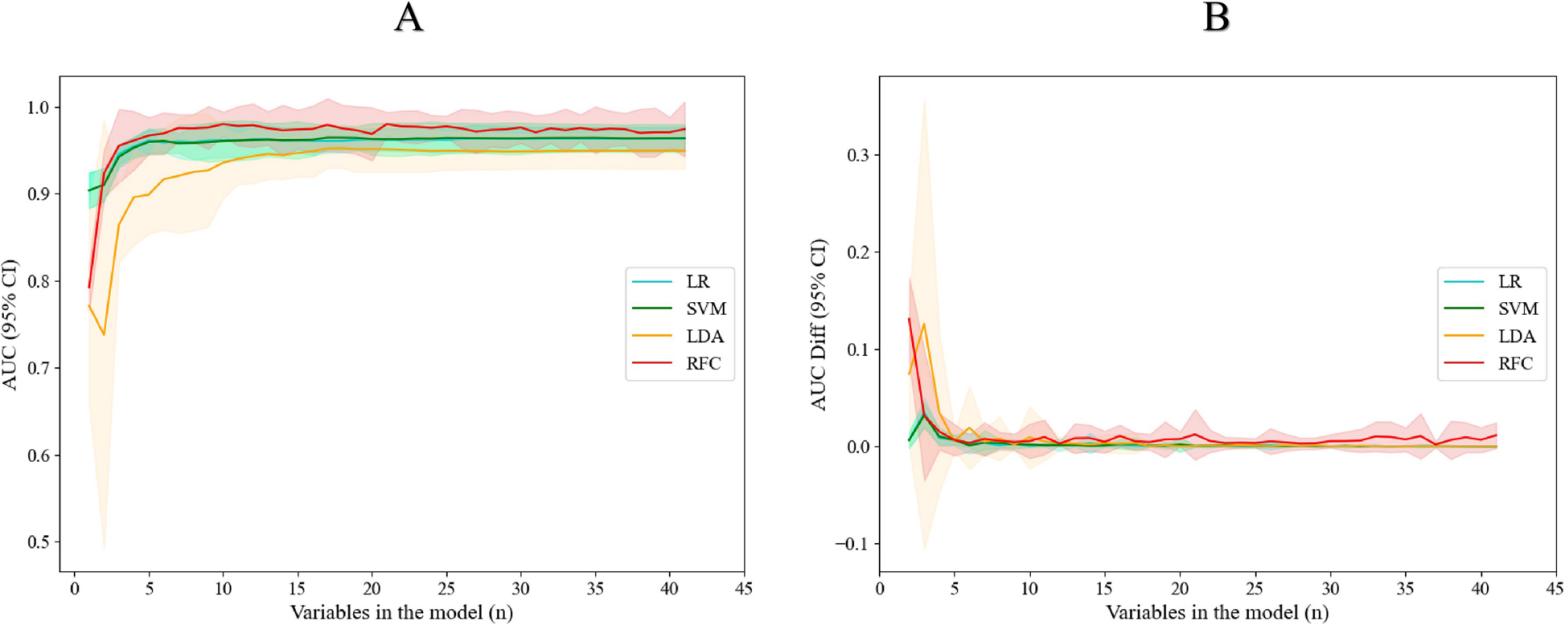
ROC analysis of the multinomial models on the training set. A shows the selection of variables for each model starting from the maximum number of variables (*n* = 67) and retaining the highest n-1 variables based on their importance in the model. B shows the ROC decline of each model as the number of features decreases, with the variables of lowest importance being gradually eliminated from the maximum number of features. ROC: Receiver Operating Characteristic, AUC: area under the receiver operating characteristic curve, LR: logistic regression, SVM: support vector machine, LDA: linear discriminant analysis, RFC: random forest classifier

As shown in Fig. 3, the RFC model performed the best among the four models. Using the recursive feature elimination method based on cross-validation, it was found that increasing the number of features above 11 did not significantly improve the AUCs of the four models, and the AUC decay rates were all lower than 0.5%. When the number of features was less than 11, the AUC decay rates of the models were almost all significantly greater than 0.5%; that is, removal of each additional feature resulted in significant loss to the predictive performance of the models. Finally, we used the top 11 feature parameters in terms of feature importance to construct a Novel IMs model. As shown in Supplementary Fig. 1, we found that four scatter plot features, D-Lym-Peak-Valley-R, D-Lym-SFL-CV, D-Lym-SFL-W and D-Lym-SFL-P were all ranked high, with D-Lym-Peak-Valley-R being the most important feature.

### Novel IMs model performance

**Fig. 4:**
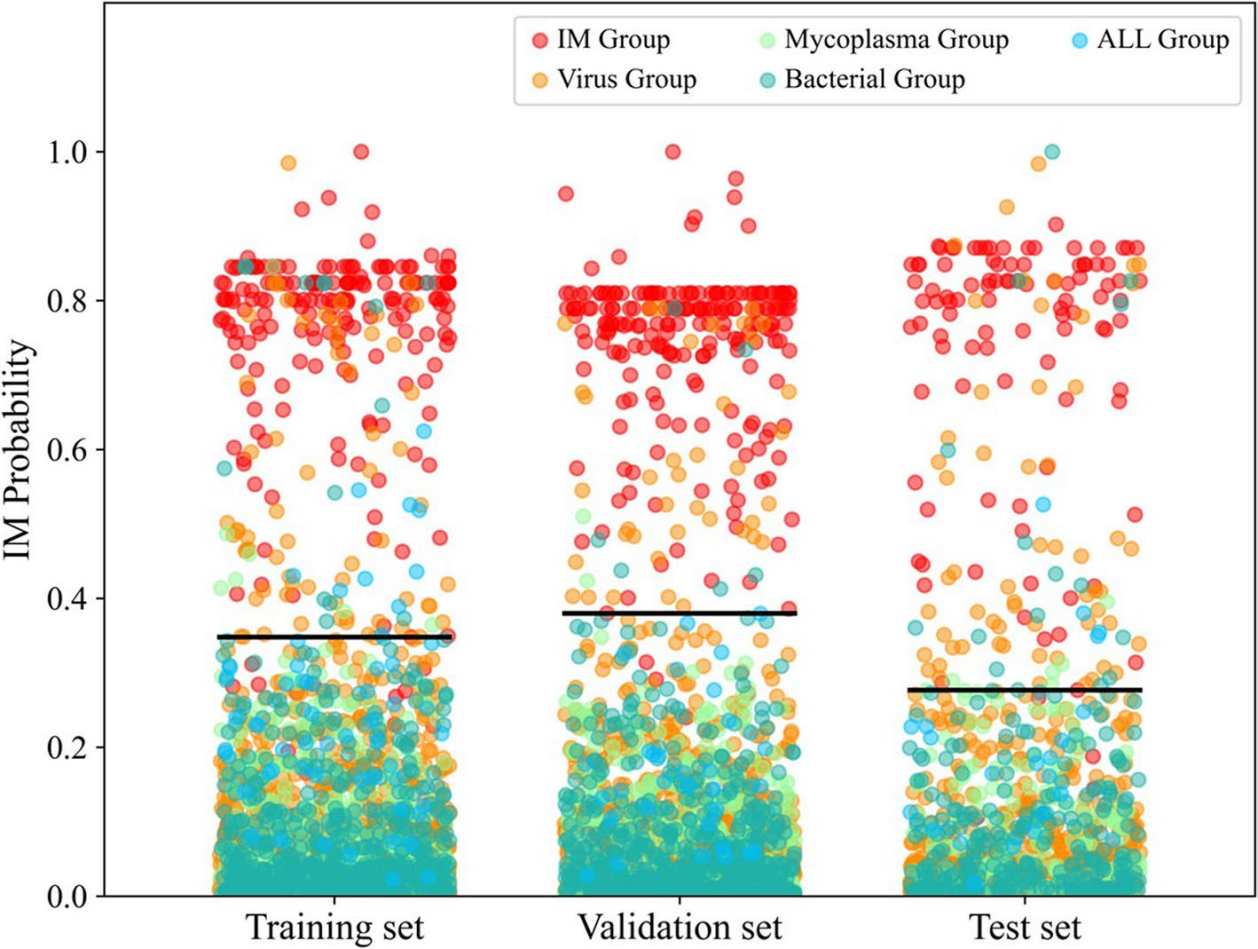
The probabilities of the Novel IMs model predicting that it is an IM for different types of samples in the training, validation, and test sets are observed from the final prediction cutoffs. IM Group: infectious mononucleosis group, Mycoplasma Group: myeoplasma pneumoniae infection group, Virus Group: virus infection group, Bacterial Group: bacterial infection group

We used the hyperparameter grid search algorithm to select the optimal hyperparameters for the established Novel IMs model, which were then Platt Scalling calibrated (Supplementary Fig. 3). The final model had excellent performance in the training set, validation set, and test set. As shown in Table 2, in identifying IM patients, the accuracy rates were all above 91.92%, the specificity rates were above 91.8%, and the sensitivity rates were all above 89.71% in the three datasets. As a screening model, the model achieves a 93.33% sensitivity for identifying IM samples within test set. The predictions of the Novel IMs model for different classes of samples in the three datasets are shown in Fig. 4. The scatter points above the histogram represents samples for which the Novel IMs model yielded high probabilities that the diagnosis was IM. The figure shows that IM patients were well differentiated from other patients, however, for a few patients with respiratory infections, we would have incorrectly predicted that they had IM.

**Table 2:** Novel IMs model performance in three sets

	**Accuracy**	**Sensitivity**	**Specificity**
Training set	94.78%	89.71%	95.30%
Validation set	97.69%	98.09%	97.64%
Test set	91.92%	93.33%	91.78%

### Comparison with models from the literature

**Fig. 5:**
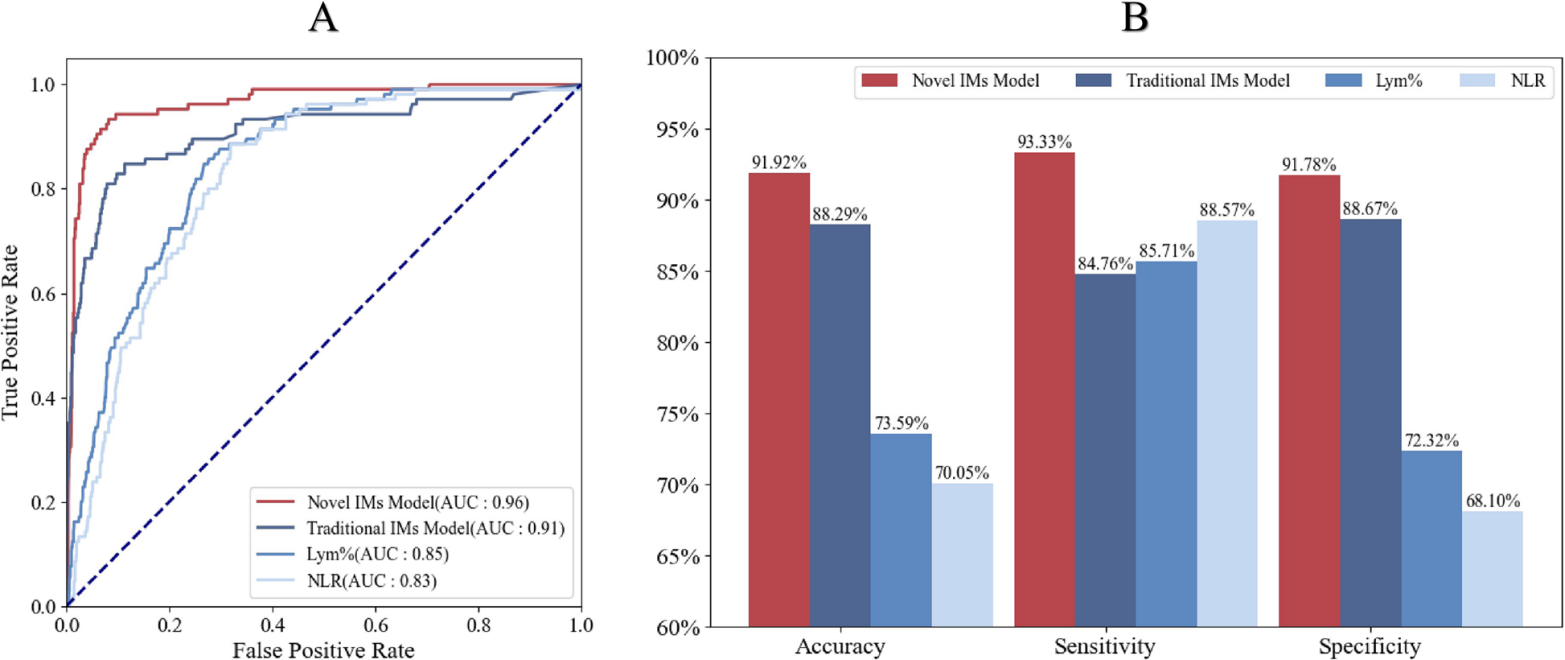
**A** Performance of different methods for infectious mononucleosis screening. **B** Receiver operating characteristic (ROC) curves of the binary decision with the area under the curve (AUC) is noted. Lym%: percentage of lymphocytes (Lym%); NLR: neutrophil-to-lymphocyte ratio

**Fig. 6:**
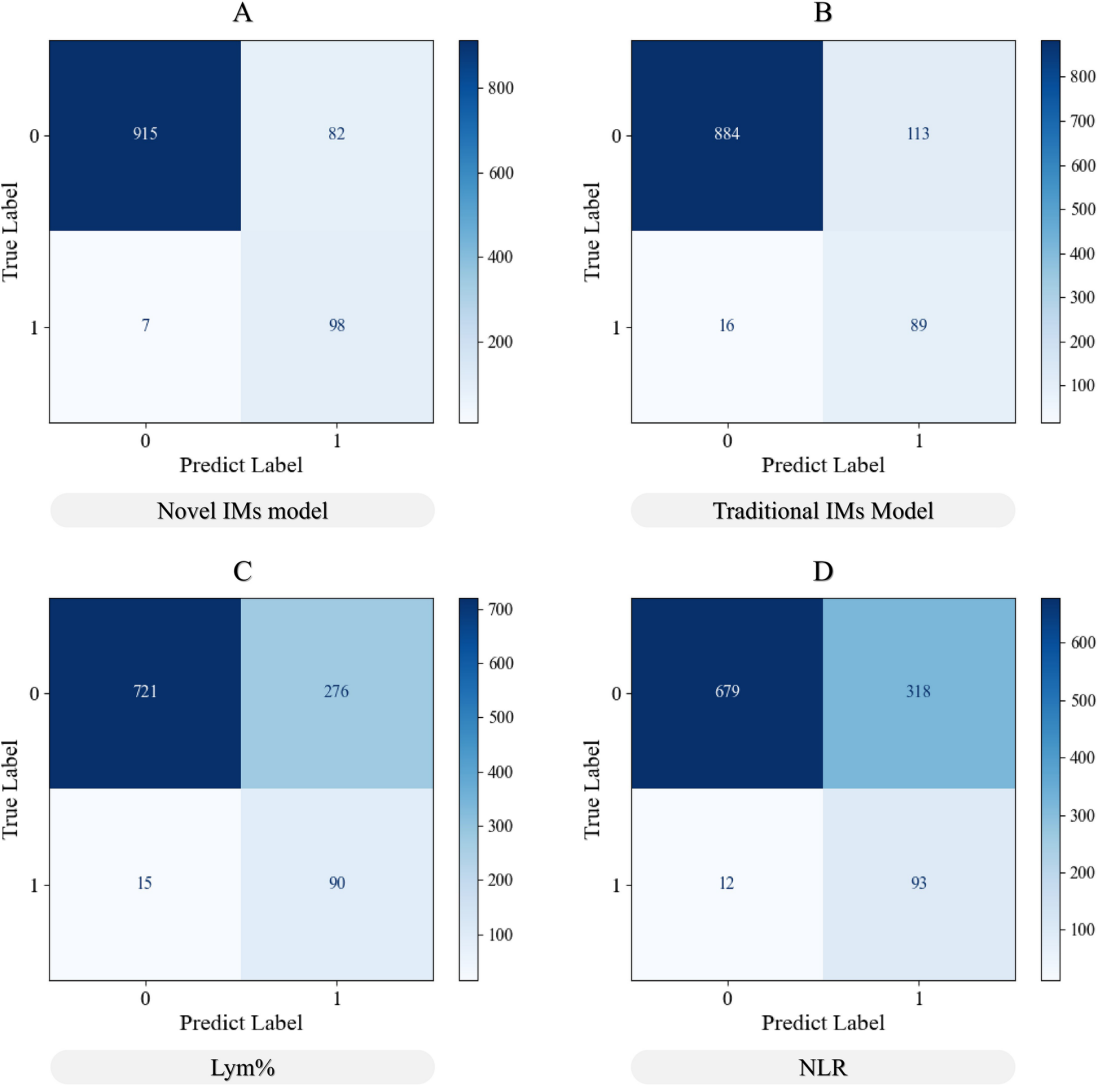
Confusion matrix of the Novel IMs model (**A**), Traditional IMs (**B**), Lym% (**C**), NLR (**D**) for screening infectious mononucleosis patients in the test set. Lym%: percentages of lymphocytes, NLR: the neutrophil-to-lymphocyte ratio

Comparing the current model with traditional clinical blood cell parameters for screening IM [6, 21, 22], the Novel IMs model had an AUC of 0.96 (Fig. 5A), an identification accuracy of 91.92%, and higher specificity and sensitivity (Fig. 5B). Especially compared with Lym% and NLR, according to an analysis of the confusion matrix (Fig. 6), the number of false-positive and false-negative samples of the Novel IMs model was lower than that of the other three methods. The screening of IM patients via the blood cell parameters LYM% and NLR alone resulted in 276 and 318 false-positive samples, respectively. The Novel IMs model reduced 318 false-positive patients (NLR) to 82. In addition, according to the DCA analysis, the net clinical benefits of the Novel IMs model were all higher than those of the other methods (Supplementary Fig. 4).

**Fig. 7:**
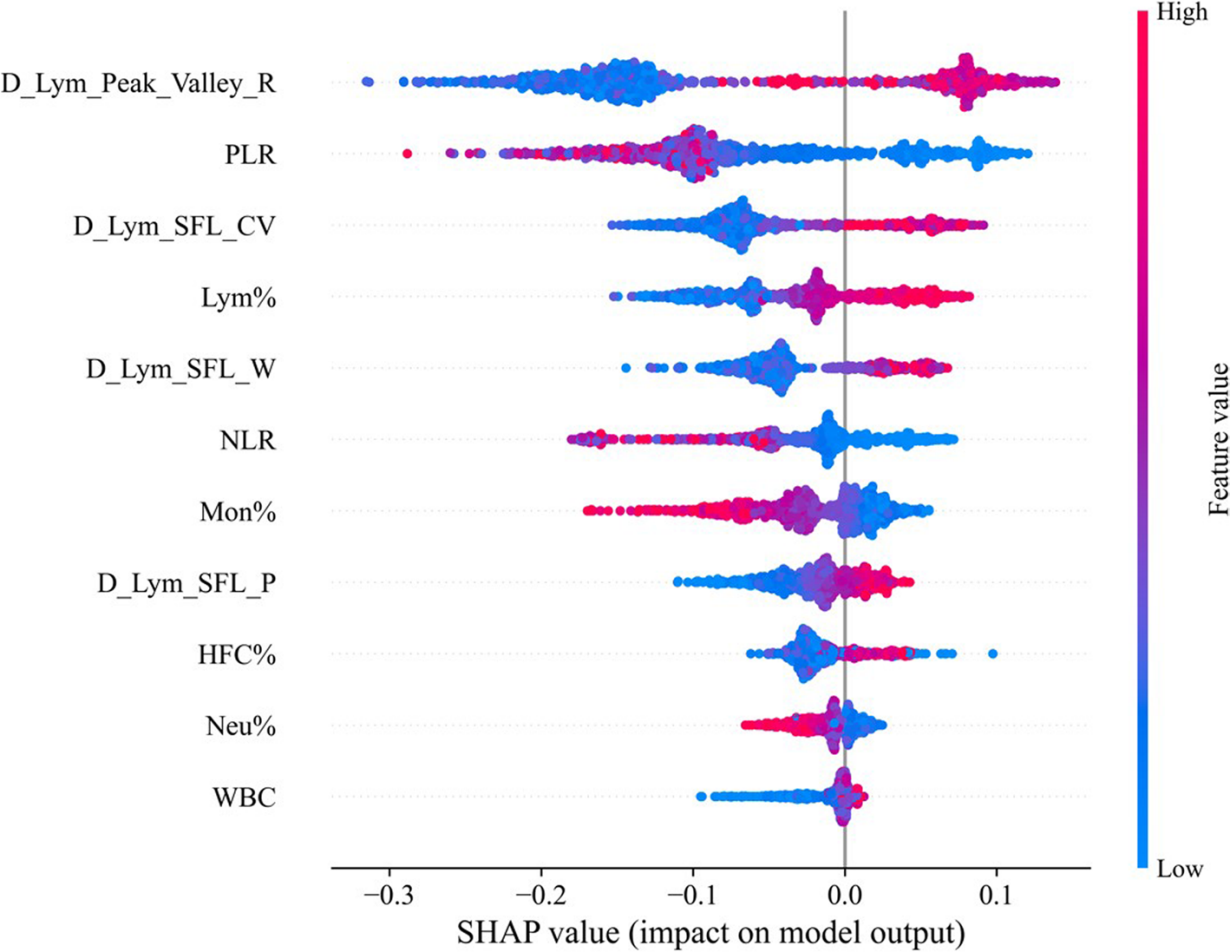
Shapley additive explanation (SHAP) summary plot of 11 features, derived by aggregating related values of a particular feature (e.g., the average, minimum, and maximum). Each dot corresponds to the SHAP value of the feature cluster for the infectious mononucleosis risk score of a given case patient or control subject at a certain point in time. A feature’s SHAP value (x-axis) represents the contribution of the specific feature to the risk score, with positive values indicating a contribution that increases the risk score and negative values indicating a contribution that lowers the score. The location of the dot on the x-axis represents its SHAP value, whereas its color represents the cluster’s value (the actual value of the feature that is represented in the cluster), with red representing higher values (for features measured along a continuum) or affirmative responses (for binary features). The dots are piled up vertically to show their density. The feature clusters are sorted by their mean absolute SHAP values

Next, we used SHAP analysis to determine the reasons for the excellent performance of the Novel IMs model. All the samples could be ranked according to the importance of the predicted features. Each dot represents a sample; red indicates that the feature value is high, blue indicates that the represents that the feature value is low, and a positive SHAP value indicates an increased risk of IM (Fig. 7). The figure shows that higher values of D-Lym-Peak-Valley-R, D-Lym-SFL-CV, D-Lym-SFL-W and D-Lym-SFL-P are associated with a higher risk of suffering from IM, and that these four parameters discriminate IM much better than PLR, NLR and HFC%.

### Variation threshold

**Fig. 8:**
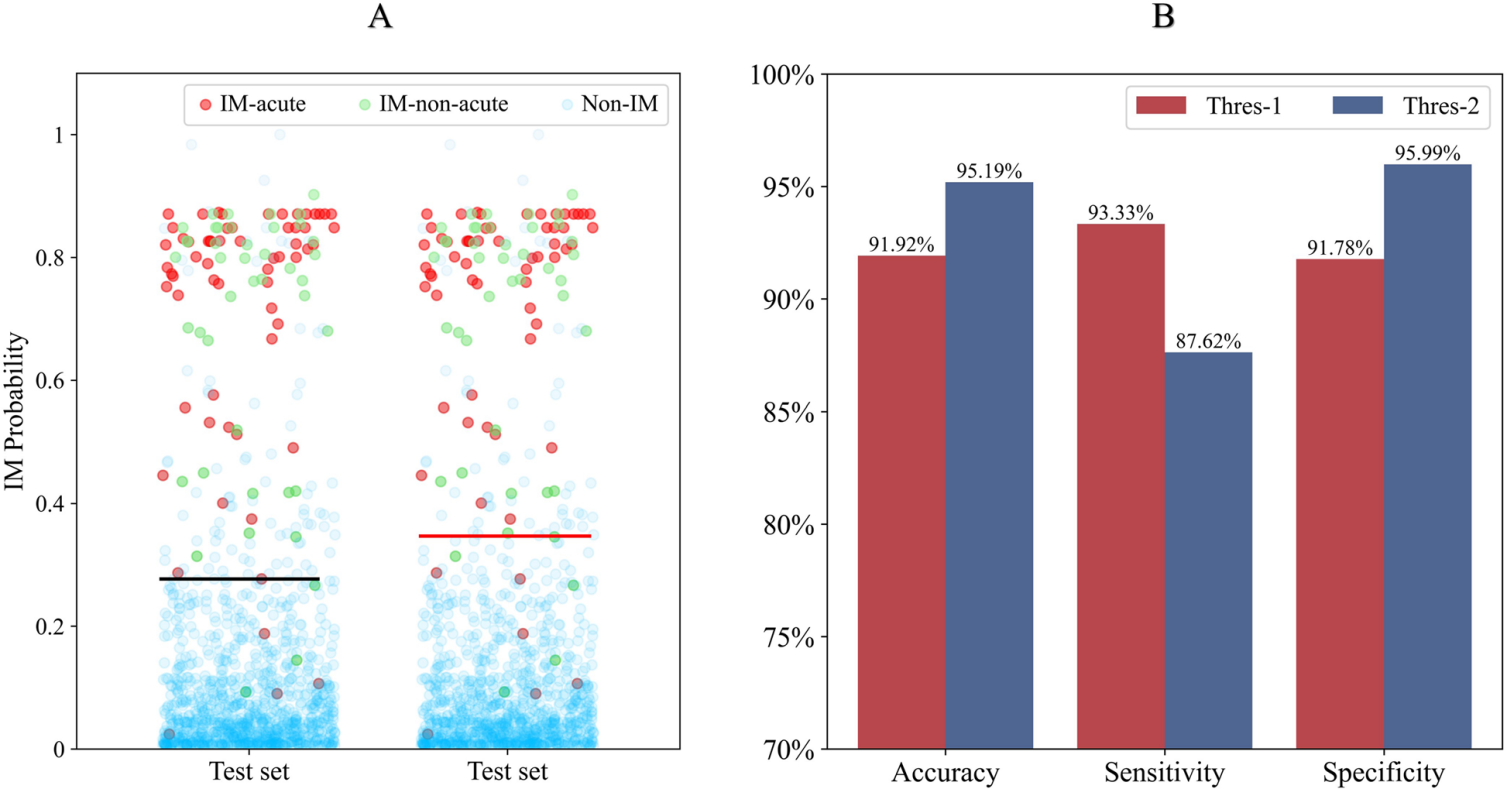
**A** shows the probability of the novel IMs model predicting an infectious mononucleosis in different types of samples in the test set from different thresholds. **B** shows the performance of different thresholds in screening for infectious mononucleosis. IM-acute: infectious mononucleosis in the acute phase, IM-non-acute: infectious mononucleosis in the nonacute phase, Non-IM: Non-infectious mononucleosis, Thres-1: Threshold 1, Thres-2: Threshold 2

We found that the changes in the blood cell scatter plot of IM patients were related to the disease stage, in which the IM patients were divided into acute-stage IM patients and nonacute-stage IM patients. The positive determination threshold of the model can be increased without affecting the model's ability to identify patients with acute-phase IM (Fig. 8). When the threshold of the Novel IMs model was increased from threshold-1 (0.53) to threshold-2 (0.65), the specificity of the Novel IMs model increased to 95.99% and the accuracy to 95.19%, but the sensitivity decreased to 87.62%, at which point the model sacrificed some identification of patients with nonacute IM.

## Discussion

Children with IM caused by primary EBV infection have significantly higher numbers of atypical lymphocytes than children with IM caused by EBV reactivation or other viruses [23] and more severe symptoms [2]. Therefore, this study established an IM screening model to identify such patients. The Novel IMs model is an FRC model based on the parameters of the 3D-DIFF scatter plot. This model can identify children with IM caused by primary EBV infection admitted to the hospital with fever after undergoing a CBC test, providing a reference for a doctor’s diagnosis. The IM screening model is an FRC machine learning model based on 3D-DIFF scattergram parameters. It can provide early indication of primary EBV-associated IM when febrile children undergo routine blood testing upon admission, helping to identify suspected IM cases for more specific confirmatory testing (such as EBV antibody or nucleic acid testing). Although some studies have shown that the diagnostic specificity of blood cell analysis with a lymphocyte percentage greater than 50% in diagnosing IM reaches 99%, the sensitivity is only 45% [11, 12]. This may be related to the immune response of patients with IM due to EBV infection and the stage of the disease. Therefore, the number of lymphocytes cannot be used to accurately identify IM. Later studies revealed that apoptotic cells appeared in IM patients [24]. Subsequently, a blood cell parameter model was established to identify apoptotic cells and thus to identify patients with IM [13–15]. However, the use of apoptotic cells to identify the IM is only valid for some IM patients [13]. The activated lymphocyte population of IM patients is composed mainly of CD8^+^ T cells and a small portion of CD19^+^ B lymphocytes (accounting for 0–2% of all lymphocytes) [23]. Peripheral T cells infected with EBV transform, and the EBV proliferates during mitosis in the body [25]. The virus-induced changes in the nuclei of T cells were reflected mainly in the 3D scatter plots of the hematology analyzer as changes in nucleic acid content, that is, signal changes in the FL direction.

Previous studies described the distribution width of the FLs of IM patients at the two-dimensional level and calculated the difference between the maximum and minimum values of lymphocyte cluster distribution [14, 15]. However, we found that even though the width of the FL distribution was increased in both IM and non-IM patients, the changes in lymphocyte clustering in the FL direction were not the same. Therefore, the changes in FLs in IM patients cannot be completely described by the distribution width alone. To describe the changes in lymphoid clusters in IM patients in detail, we obtained a three-dimensional lymphatic cluster probability density distribution through the number of cells under the FL value and calculated D-Lym-SFL-W, D-Lym-SFL-P, D-Lym-SFL-CV and D-Lym-SFL-Peak-Valley-R, comprehensively characterizing the changes in the distribution of lymphoid clusters in the low FL direction for IM patients. These characteristic parameters were combined with 41 relevant blood cell analysis parameters, which were then subjected to recursive feature elimination to construct the optimal IMs model. Ultimately, we selected 11 parameter features, among which D-Lym-Peak-Valley-R was the most important feature parameter; this parameter describes whether the FL signal of IM patients is continuous and may be related to changes in viral proliferation in lymphocyte nuclei. At a decision threshold of 0.53, the Novel IMs model distinguished IM patients in the training set, validation set, and test set well, but it also misidentified some patients with viral infections. When compared with the NLR, LYM% and Traditional IMs model, the Novel IMs model, combined with the characteristic parameter CPD of the DIFF channel—which best described the development of lymphatic clusters in the FL direction—achieved the best performance in terms of AUC, sensitivity, specificity, and small numbers of false-positive and false-negative samples. The Novel IMs model was further explained by SHAP analysis, which showed that the four parameters D-Lym-SFL-W, D-Lym-SFL-P, D-Lym-SFL-CV and D-Lym-SFL-Peak-Valley-R were the important in quantifying FL fluorescence according to the probability density.

Further analysis of the false-negative samples identified by the Novel IMs model revealed that 6 of the 9 were nonacute IM patients. We found that the scatter plot of the peripheral blood parameters of the patients changed with the stage of EBV infection. In the 3D-DIFF scatter plot of IM patients in the nonacute stage, the number of lymphoid clusters distributed in the high-FL direction in the 3D-DIFF scatter plot was lower than that of patients in the acute stage. Therefore, Threshold-1 represents our recommended cutoff, while Threshold-2 is provided as an adjustable alternative that clinicians may use based on their assessment of the patient's disease stage. Analysis of 82 false-positive samples from the Novel IMs model revealed that 75% of them were patients infected with RSV or ADE. This is mainly because when phagocytes “clear” virus-infected cells, more double-helical RNA is released in the body, eventually increasing the total double-helical RNA concentration in lymphocytes or granulocytes [26]. The nucleic acid content of lymphocytes in patients with viral infection was altered, which manifested as changes in lymphocyte clusters in the FL direction on the 3D-DIFF plot in a manner similar to the changes observed in IM patients. If follow-up prospective studies can incorporate EBV nucleic acid test results correlated with fluorescence changes in IM patients, new findings may emerge. Currently in order to solve this problem, we used peripheral blood cell morphology to determine differences between IM patients and patients with viral infection. We identified all lymphocytes and reactive lymphocytes from the peripheral blood morphological results of 15 IM patients and 15 patients with viral infection. At the single-cell level, we refined the visualization of the cytoplasm and nucleus and measured the volumes of the cell/nucleus/cytoplasm (Supplementary Fig. 2). The results revealed that in the peripheral blood lymphocytes of IM and virus-infected patients, the nucleus-to-cytoplasm ratios were 2.68 ± 0.51 and 3.34 ± 0.37, respectively, with significant differences. To further increase the differentiation effect of the Novel IMs model between virus-infected patients and IM patients, the results of cell morphology, that is, characteristics reflecting the nucleus-to-cytoplasm ratio of lymphocytes, can be incorporated into the model. However, the IM screening model established in this study did not include the relevant morphological features in an attempt to ensure relatively broad applicability of the model. This is an important advantage of building a model on the basis of blood cell scatter plots. Blood cell analysis at the doctor's visit can immediately suggest IM, avoiding the need for morphological examination. The rapid acquisition of blood cell analysis parameters allows the results of this model to be used for the early indication of infection and may influence clinical decision-making.

The Novel IMs model in this study is based only on blood cell analysis parameters. It can identify IM through a simple CBC, which is very helpful for diagnosing IM, which is otherwise difficult to identify even for experienced doctors [27]. Compared with the detection of EBV antibodies or nucleic acids, the Novel IMs model in this study can reduce the burden on patients, improve the coverage of IM screening, reduce medical costs, enable IM patients to receive supportive treatment as soon as possible, reduce the occurrence of rash due to taking moxicillin, and prevent the development of serious complications, such as splenic rupture, caused by IM [3, 6]. Notably, in the Novel IMs model established in this study, the Mindray BC-7500 CRP Hematology Analyzer provides a comprehensive view of the morphological status of white blood cells through scatter plot parameters obtained by laser flow technology and fluorescent multidimensional staining. However, hematology analyzers from different manufacturers will output parameters based on different calculations and thresholds, severely limiting the extensive clinical application of ML models constructed on the basis of such parameters. This problem must be solved if model applicability is to be improved [28]. The reproducibility of this model requires more extensive validation through multiple approaches, particularly via prospective clinical trials, to fully establish its effectiveness as an IM screening tool. Such validation should also evaluate potential cost and labor savings enabled by the model implementation. Finally, the ML model can only assist the doctor in the preliminary screening of the IM, and not every patient can be perfectly identified [29]; it is still the doctor who ultimately makes the next step of clinical diagnosis and treatment. Although the construction of the ML model based on blood cell parameters in the present study is limited, it is particularly important for febrile children. Febrile children often cannot accurately describe their symptoms, making it difficult for clinicians to establish a clear diagnosis. When only fever is present without specific symptoms, physicians typically face a wide range of initial test options. Moreover, these patients may show similar peripheral blood patterns. In such cases, a blood cell parameter-based model could serve as the clinician's best tool to guide more targeted diagnostic and therapeutic decisions.

## Summary

In this study, we innovatively constructed a Novel IMs model based on 3D-DIFF scattergrams to observe the changes of lymphatic particle clusters and further analyzed the scattergram parameters to quantify the changes of fluorescence signals in IM patients through probability density distribution plots. Compared with other indicators, the Novel IMs model had higher accuracy and better performance. This study was the first to employ blood cell analysis parameters for the construction of a model that can be used to screen IM patients from among febrile children at low cost. Blood cell analysis can provide convenient and low-cost initial screening of IM, addressing the difficulty clinicians have in diagnosing the disease, improving screening coverage, and further reducing misdiagnoses and missed diagnoses.

## Supplementary Information

Supplementary Material 1

Supplementary Material 2

Supplementary Material 3

Supplementary Material 4

## Data Availability

The raw data can be obtained on request from the corresponding author. The email is yugbme@zju.edu.cn.
