# Supplementary figures and images for: Novel screening model for infectious mononucleosis in febrile pediatric patients using a 3D-DIFF scattergram
Source: BMC Infect Dis. 2025 Nov 7;25:1522. doi: 10.1186/s12879-025-11662-3 (PMC12595630; doi:10.1186/s12879-025-11662-3)

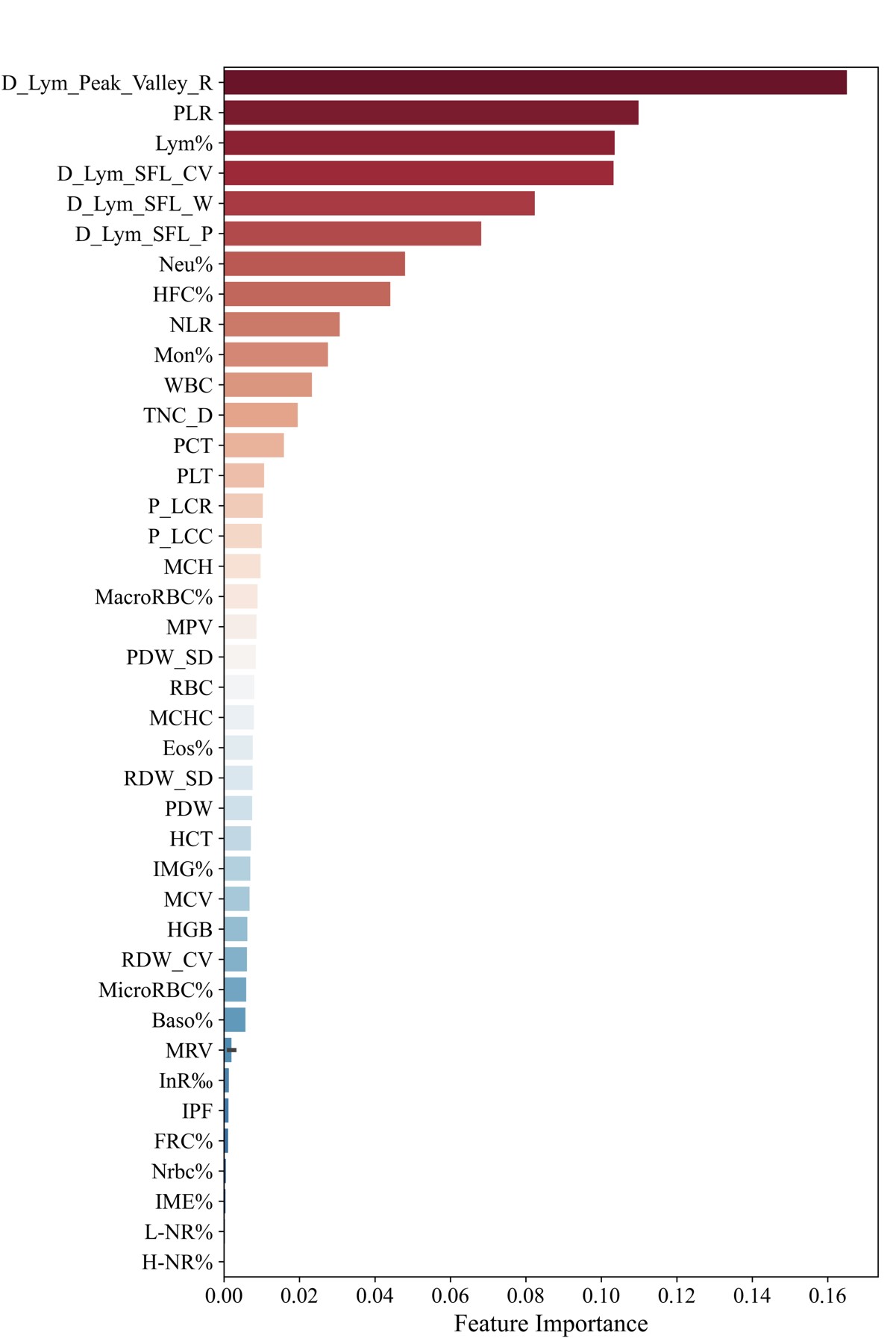

Supplement: Supplementary file 1 — Supplementary Material 1 [file 12879_2025_11662_MOESM1_ESM.jpg]

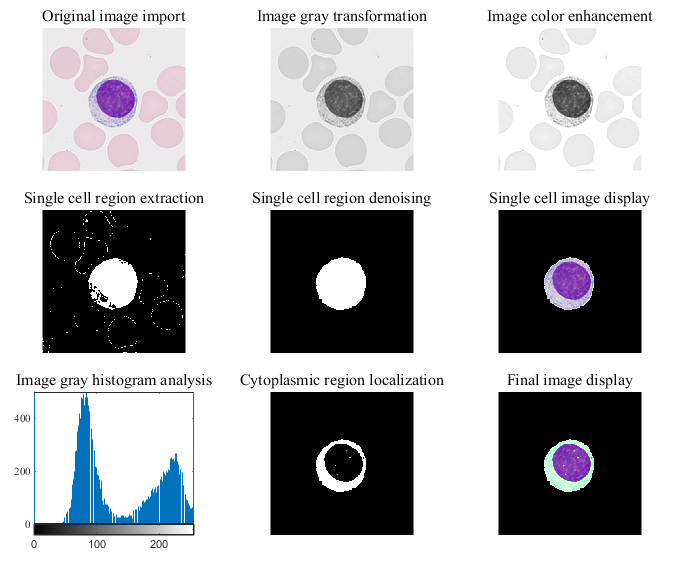

Supplement: Supplementary file 2 — Supplementary Material 2 [file 12879_2025_11662_MOESM2_ESM.tif]

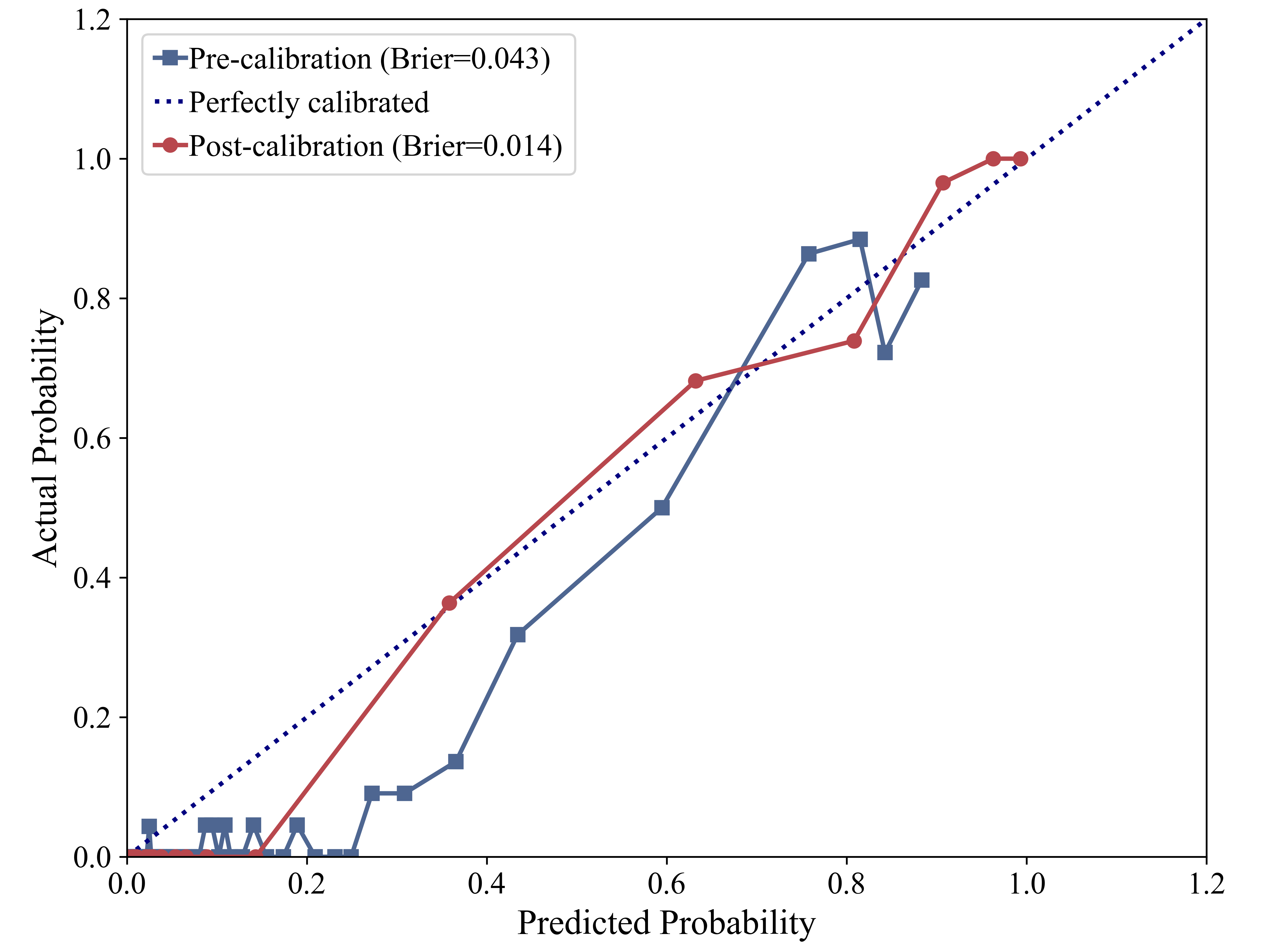

Supplement: Supplementary file 3 — Supplementary Material 3 [file 12879_2025_11662_MOESM3_ESM.png]

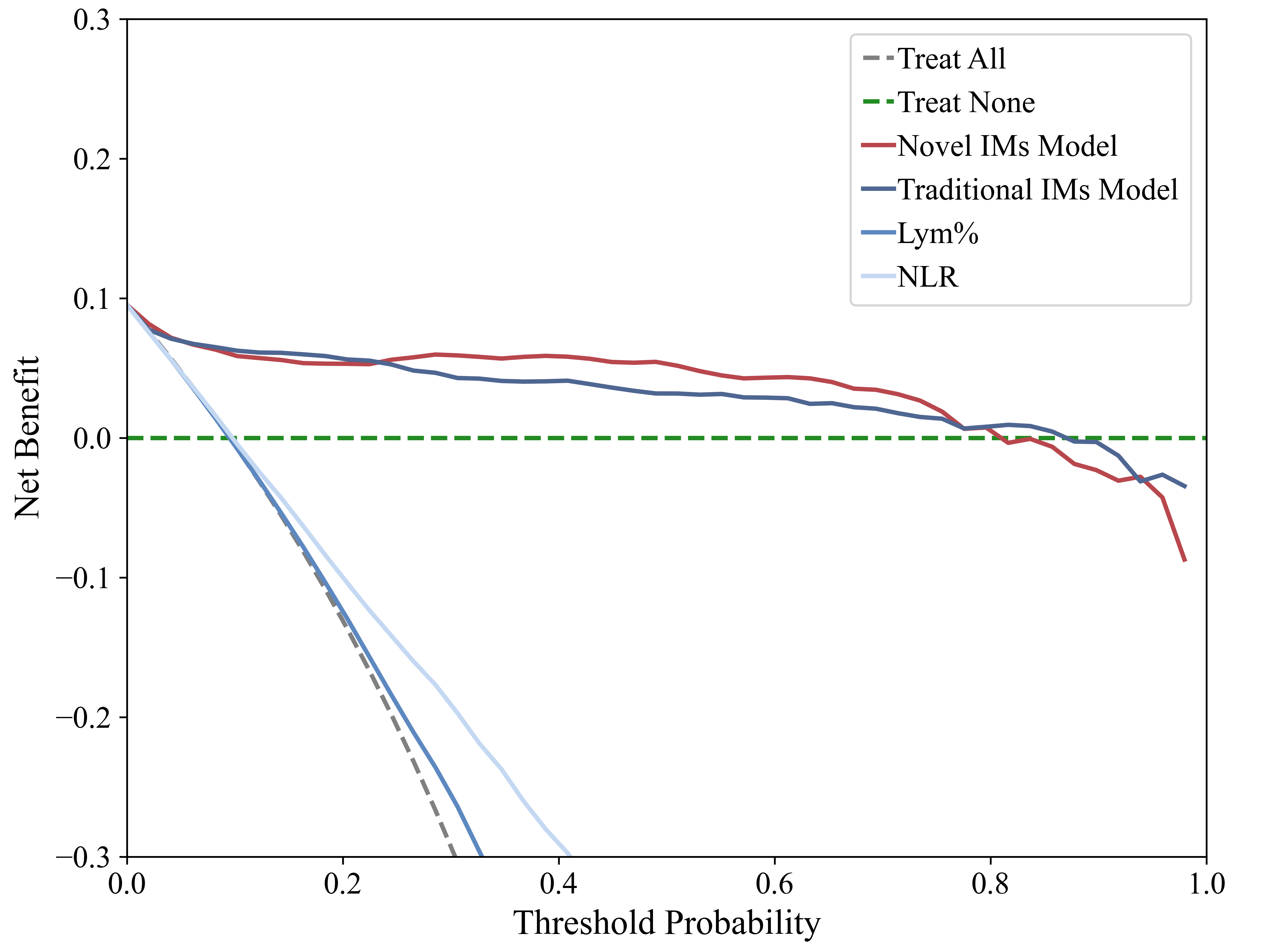

Supplement: Supplementary file 4 — Supplementary Material 4 [file 12879_2025_11662_MOESM4_ESM.png]
